# Exercise capacity and cardiac function in adolescents born post-term

**DOI:** 10.1038/s41598-018-31343-3

**Published:** 2018-08-28

**Authors:** Mrinal Murali, Paul L. Hofman, José G. B. Derraik, Wayne S. Cutfield, Tim Hornung, Silmara Gusso

**Affiliations:** 1 Liggins Institute, University of Auckland, Auckland, New Zealand; 2 A Better Start – National Science Challenge, University of Auckland, Auckland, New Zealand; 3 Department of Women’s and Children’s Health, Uppsala University, Uppsala, Sweden; 4 Department of Paediatric Cardiology, Starship Children’s Hospital, Auckland, New Zealand

## Abstract

There is some evidence that children born post-term (≥42 weeks of gestation) have metabolic abnormalities that may be associated with an increased risk of adverse health outcomes in adulthood. However, there are no data as to whether adolescents born post-term display alterations in aerobic capacity or cardiovascular function. We studied 48 adolescents (56% males) in Auckland (New Zealand) with a mean age of 14.3 years (SD = 1.7): 25 born post-term and 23 born at term (37–41 weeks of gestation). Assessments included metabolic markers in blood, whole body DXA scans, 24-hour ambulatory blood pressure monitoring, maximal exercise capacity, as well as cardiac MRI scan at rest and during submaximal exercise. Exercise capacity was lower in the post-term than in control participants (44.5 vs 47.8 ml/kgffm/min; p = 0.04). There were no differences in left ventricular volumes at rest and during exercise between groups. The 24-hour ambulatory blood pressure monitoring also showed no differences between the two groups. Being born post-term was associated with reduced exercise capacity, but with no observed differences in central cardiac function. We speculate that the reduction in exercise capacity may be due to changes in the peripheral vascular system.

## Introduction

Post-term birth, defined as birth on or after 42 weeks of gestation, has been shown to be associated with an increased risk of morbidity and mortality, both in the short- and long-term^1,2^. As the risk of delivery complications and neonatal morbidity climb rapidly once pregnancies go past 41 weeks of gestation, there are universal international recommendations to avoid post-term delivery^3^. Although obstetric practice has changed, the rates of post-term birth vary considerably worldwide, ranging from 0.4% of live births in Austria to as many as 8.1% in Denmark^4^.

Currently, there is little to no research involving the later impact of being born post-term. The vast majority of the research has focused on a narrow window, i.e. on the increase risk of perinatal complications^1,5^. Only relatively recently post-term birth has been identified as a risk factor for adverse metabolic outcomes in childhood and adolescence^6,7^. Post-term children displayed similar metabolic and body composition abnormalities compared to preterm and small for gestational age (SGA) children with reduced insulin sensitivity, higher fat mass, and higher blood pressure^6,7^. In addition, Derraik *et al*. showed that post-term birth was associated with greater BMI and increased risk of overweight and obesity in adult women, particularly among those born ≥43 weeks gestation^8^. There are a number of factors that may be associated with an increased risk of post-term delivery^5^. Some of these are potentially modifiable, notably maternal obesity^9^.

Previous studies investigating growth and metabolism in children born post-term^6,7^ showed a 34% reduction in insulin sensitivity in pre-pubertal children and an increased risk of obesity in adolescence males. These abnormalities are similar to those observed in children and adults born preterm or SGA, suggesting post-term children may have similar metabolic sequelae^10–13^. Children born SGA and preterm have also been shown to have impairments in cardiovascular function and aerobic capacity^2,14–18^. Preterm children have reduced aerobic capacity compared to term counterparts with higher rates of hypertension and increased left ventricular mass and volume^2,14–18^. SGA infants also display cardiovascular changes with more globular hearts, impaired relaxation, increased vascular wall thickness, and higher blood pressure compared to controls born of appropriate weight-for-age^19^. Adults born both preterm and SGA have increased risk of cardiovascular disease suggesting that abnormalities detected in childhood progress to clinically relevant conditions over time^14^. Thus, childhood alterations in cardiac structure and function may be indicative of later adult disease.

There is currently no information regarding the impact of being born post-term on metabolic and cardiovascular health later in life, particularly during adolescence. This is important considering that a reduction in insulin sensitivity has been shown to impact cardiac function and exercise capacity in young individuals^20^. Insulin resistance and hyperinsulinemia are universal in all adolescents during puberty, and are primarily a result of the rise in sex steroid and growth hormone levels^21^. Increased insulin secretion is required to maintain euglycaemia^22^. If this is transient there are no sequelae and indeed it may promote anabolism during puberty. However, a persistent reduction in insulin sensitivity leads to insulin resistance and an increase in cardiovascular risk, stroke and type 2 diabetes mellitus in adulthood^23^. The heart is an insulin-responsive organ, and the presence of insulin resistance and obesity (as previously demonstrated in post-term children) could underpin the altered cardiac function. A study with 25,618 adults born post-term^24^ showed no differences in maximal exercise capacity in adulthood compared to subjects born at term, but data from the study were not adjusted for metabolic tissue (lean mass) and did not include cardiac function assessments. Therefore, we aimed to assess whether aerobic capacity and cardiovascular function in healthy adolescents born post-term was altered compared to those born at term.

## Methods

### Participants

Potential post-term participants (≥42 weeks gestation) born between 2000 and 2001 were identified from the obstetrics database at the National Women’s Health (Auckland City Hospital), which contains data on births from three hospitals throughout the Auckland area (New Zealand). Each recruited participant born post-term was asked to invite up to two friends or family members who were born at term (38–41 weeks of gestation), so that participants in both groups were approximately matched for age, sex, and lifestyle, as well as reported physical activity levels. Participants self-reported as being physically active or not, which was defined as ≥3 hours a week of guided exercise. This information was obtained using a questionnaire^6,25,26^.

Participants were included if they were able to perform vigorous exercise on a bike, did not suffer from claustrophia, and able to perform exercise while lying down during the MRI scan. Participants were excluded if they had evidence or history of musculoskeletal, cardiovascular or pulmonary disease; used cardiovascular or hypertensive medication; had any contraindication for MRI scanning including pacemakers, metal fragments in the eye, spinal column stimulators, aneurysm clips in the brain; were pregnant; were born small for gestational age (birth weight below the 10^th^ percentile for the gestational age and sex), large for gestational age (birth weight above the 90th percentile for that gestational age and sex), or following assisted reproduction; if born from mothers that had gestational diabetes, pre-eclampsia, gestational or pre-existing hypertension, chronic illness, or maternal recreational drug use during pregnancy (including tobacco).

Socioeconomic status was assessed using the overall Index of Multiple Deprivation (IMD), which encompasses deprivation across seven domains (i.e. Access, Crime, Education, Employment, Health, Housing, and Income)^27^.

Ethics approval was given by the Health and Disability Ethics Committees (Ministry of Health, New Zealand – 14/NTA/15). All participants provided written and verbal informed consents, while those aged <16 years also needed written parental consent. This study was performed in accordance with all appropriate institutional and international guidelines and regulations for medical research, in line with the principles of the Declaration of Helsinki.

### Assessments

All clinical assessments were carried out at the Liggins Institute in two separate visits to our clinic. In the first visit, height was measured to nearest mm using a Harpenden stadiometer. Weight and body composition were determined by dual-energy x-ray absorptiometry (DXA; Lunar Prodigy 2000; General Electric, Madison, USA). Physical activity was assessed by questionnaire, while birth weight was extracted from hospital records. Fasting venous blood samples were drawn to assess lipid profile, as well as glucose, insulin, IGF-I, IGFBP-1, leptin, and adiponectin concentrations. Lipids and glucose were measured on a Roche Hitachi 902 autoanalyser (Hitachi High Technologies Corporation, Tokyo, Japan) and insulin on a Roche Elecsys 2010. Commercially available ELISA kits were used to analyse leptin, adiponectin, IGF-I, and IGFBP-1.

For the assessment of Maximal Exercise Capacity ($\dot{{\rm{V}}}$O_2max_), participants pedaled to exhaustion on an electronically-braked upright cycle ergometer (Schiller CG6340 BAAR-Switzerland) with simultaneous measurements of inspired O_2_ and CO_2_ volumes (ParvoMedics TrueOne 2400 Metabolic Measurement system, Parvomedics; Sandy, Utah, USA), based on an established protocol using an upright cycle ergometer^26,28^. In brief, participant’s initial workload was set at 55 watts, with an increase of 15 watts every minute. Participants were advised to remain at or above 60 revolutions per minute throughout the test. The protocol was designed to last a maximum of 15 minutes, and the test was terminated when the participant was unable to continue due to exhaustion, pain, discomfort, or technical error. The participant’s maximum exercise capacity was determined by the average of the two highest consecutive VO_2_ values. Heart rate and workload for each participant was recorded every minute during the test. Blood pressure was measured after resting for 5 minutes prior to the test and immediately after the test termination.

At the end of the first visit, participants were fitted with an oscillometric device (Spacelabs 90217; Spacelabs Medical Inc, Redmond, Washington, USA) on the non-dominant arm for the 24-hour ambulatory blood pressure monitoring. Blood pressure was measured every 20 minutes 07:00–22:00 and every 30 minutes 22:00–07:00 over 24 hours.

Participants returned to our clinic within a week of the first visit, when cardiac structure and function were measured using MRI scans. Left ventricular (LV) structure and function were assessed at rest and during submaximal exercise using a 1.5 Telsa Magnetom Avanto MRI scanner (Siemens Erlangen, Germany), and an MRI-compatible cycle ergometer as previously described^26,29^. In brief, cardiac MRI images were obtained during mid expiration breath-hold for 5–10 seconds. Six short-axis slices from the base to the apex of the LV and 3 long-axis slices were obtained with 2 slices per breath-hold with a 6-mm slice thickness. Images were acquired at rest and during submaximal exercise. The latter were obtained once one minute of targeted steady-state heart rate (110 ± 5 beats per minute) was reached. The power (watts) or work rate at which the subject exercised was also recorded. Cardiac MRI images were analysed using the Cardiac Image Moddeller software (Auckland, New Zealand) to obtain left ventricular mass, ejection fraction, stroke volume, and end-diastolic and end-systolic volumes. Cardiac output was calculated by multiplying heart rate by stroke volume. Parameters were also indexed for participant’s fat-free mass.

### Sample size and power calculation

Power calculation was based on data from a previous study on non-obese adolescents aged 16.7 years on average, with a standard deviation for VO_2max_ indexed for fat-free mass of 1.4^26^. A study with 20 controls and 20 adolescents born post-term was powered to detect a difference in VO_2max_ indexed for fat-free mass of ±1.27 ml/kg FFM/min between groups, with 80% power and α = 0.05. To account for an approximate 10% loss due to dropouts, we aimed to recruit 22 participants per group. The same sample size (i.e. 20 per group) was powered to detect differences in stroke volume and left ventricular mass of ±0.06 ml/kg FFM and ±0.07 g/kg FFM between groups, respectively.

### Statistical Analysis

Demographic data for post-term and control groups were compared with one-way analyses of variance and Chi-square tests. Outcomes were compared using linear mixed regression models, adjusting for sex and age, and including family as a random factor to account for sibling clusters. Statistical analyses were carried out in Minitab v.16 (Pennsylvania State University, State College, PA, USA) and SAS v.9.4 (SAS Institute, Cary, NC, USA). All statistical tests were two-tailed and maintained at a 5% significance level.

## Results

**Figure 1:**
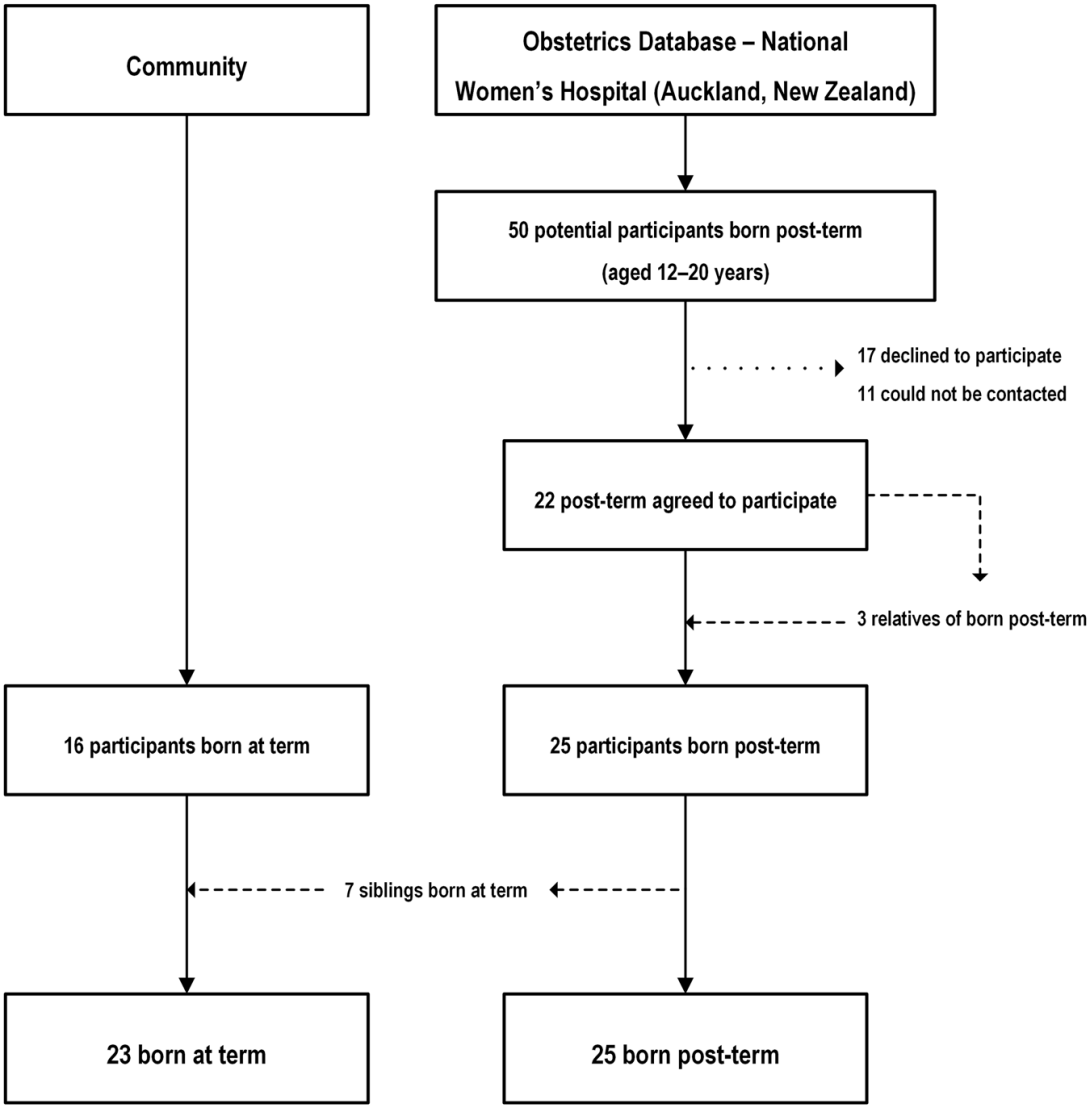
Flow diagram describing the recruitment of participants into the study.

There were fifty post-term adolescents in the National Woman’s Health database who met the study criteria. From those, 17 declined to participate and 11 were unable to be contacted. Three post-term participants had post-term siblings/family members that met our inclusion criteria and were also invited to the study. Hence we recruited 25 post-term participants. Twenty six term-born control adolescents were also recruited. Seven were term born siblings of post-term participants and 19 from the community, family and friends (Fig. 1). There were no observed differences in levels of socioeconomic deprivation or of self-reported physical activity (data not shown).

Participant’s age, birth weight SDS, gender ratio, ethnic composition, blood variables and BMI were similar between groups (Table 1). Birth weight in grams was higher in the post-term group compared to term born group (Table 1). Percentage body fat and fat-free mass assessed by DXA scan showed no differences between the two groups (Table 1). Maximal exercise capacity parameters including heart rate, systolic and diastolic blood pressure, workload and $\dot{{\rm{V}}}$O_2max_ (L/min) showed no differences between groups. However, when normalized for fat-free mass the post-term group showed lower exercise capacity (Table 2). Cardiac parameters assessed at rest and during submaximal exercise using MRI technology revealed no differences between groups (Table 3). The 24-hour ambulatory blood pressure monitoring are shown on Table 4. Again, there were no differences between groups.

**Table 1:** Baseline characteristics of participants.

		Term	Post-term	*P*-value
n (% female)		23 (43%)	25 (44%)	0.97
Demography	Age (years)	14.3 ± 0.4	14.2 ± 0.3	0.95
	Ethnicity (New Zealand European)	96%	84%	0.17
	Birth weight (g)	3552 ± 85	3938 ± 101	**0.006**
	Birth weight SDS	0.35 ± 0.18	0.45 ± 0.21	0.73
	Gestational age (weeks)	39.3 ± 0.2	42.1 ± 0.1	**0.001**
Anthropometry	Weight (kg)	57.3 ± 2.1	55.6 ± 2.0	0.53
	Height (m)	165.2 ± 1.5	166.1 ± 1.5	0.64
	BMI (kg/m^2^)	20.3 ± 0.6	20.3 ± 0.6	0.97
Body composition	Total body fat (%)	24.6 ± 2.0	23.0 ± 1.9	0.54
	Android fat to gynoid fat ratio	0.76 ± 0.04	0.76 ± 0.04	0.99
	Fat-free mass (kg)	42.7 ± 1.2	42.5 ± 1.1	0.85
Blood analysis	HDL cholesterol (mmol/L)	1.6 ± 0.1	1.6 ± 0.1	0.64
	LDL cholesterol (mmol/L)	2.6 ± 0.1	2.6 ± 0.1	0.95
	Total cholesterol (mmol/L)	4.6 ± 0.2	4.4 ± 0.2	0.28
	Fasting insulin (uIU/ml)	11.7 ± 1.2	12.2 ± 1.2	0.76
	Fasting glucose (mmol/L)	5.0 ± 0.1	5.0 ± 0.1	0.87
	Leptin (ng/ml)	7.0 ± 1.2	6.7 ± 1.2	0.84
	Triglycerides (mmol/L)	0.9 ± 0.1	0.8 ± 0.1	0.31
	IGF-I (ng/ml)	212 ± 15	222 ± 15	0.61
	Adiponectin (ng/ml)	14196 ± 1182	12788 ± 1133	0.39

Data are means ± standard errors, with anthropometric and body composition data adjusted for sex and age. P-values are shown in bold for statistically significant comparisons (at p < 0.05).

A/G ratio, android fat to gynoid fat ratio; BMI, body mass index; HDL, high-density lipoprotein; IGF-I, insulin-like growth factor I; LDL, low-density lipoprotein; SDS, standard deviation score.

**Table 2:** Baseline functional exercise capacity among participants born post-term and at term.

		Term	Post-term	*P*-value
At rest	Heart rate (bpm)	91 ± 2	94 ± 2	0.41
	Diastolic blood pressure (mmHg)	75 ± 1.5	74 ± 1.5	0.56
	Systolic blood pressure (mmHg)	116 ± 1	116 ± 1	0.88
	Mean arterial pressure (mmHg)	89 ± 1	88 ± 1	0.64
**Maximal exercise**	Heart rate (bpm)	185 ± 2	185 ± 2	0.92
	Diastolic blood pressure (mmHg)	57 ± 1	61 ± 1	0.12
	Systolic blood pressure (mmHg)	152 ± 2	148 ± 2	0.14
	Mean arterial pressure (mmHg)	104 ± 1	104 ± 1	0.92
	VO_2_ max (L/min)	36.6 ± 1.1	34.5 ± 1.1	0.19
	VO_2_ max (ml/kgffm/min)	47.8 ± 1.2	44.5 ± 1.2	**0.044**
	Workload (W)	172 ± 6	173 ± 6	0.85

Parameters were measured at rest and during VO_2max_ testing on a stationary cycle. Data are means ± SEM, adjusted for sex and age. P-values are shown in bold for statistically significant comparisons (at p < 0.05).

VO_2,_ rate of oxygen consumption.

**Table 3:** Cardiac parameters at rest and during submaximal exercise among participants born post-term and at term.

	REST			EXERCISE		
	Term	Post-Term	*P*-value	Term	Post-term	*P*-value
Heart rate (beats/min)	72 ± 2	74 ± 2	0.64	114 ± 1	116 ± 1	0.21
LVM (g)	98 ± 3	99 ± 3	0.81	—	—	
LVM (g/kgffm)	2.3 ± 0.1	2.3 ± 0.1	0.89	—	—	
Ejection fraction (%)	59.9 ± 1.0	61.8 ± 0.9	0.18	63.1 ± 1.6	64.9 ± 1.6	0.33
CO (l/min)	5.8 ± 0.3	6.2 ± 0.3	0.35	9.0 ± 0.4	9.6 ± 0.4	0.22
CO (ml/min/kgffm)	141.7 ± 7.4	139.7 ± 7.1	0.84	221.9 ± 10.2	227.5 ± 9.9	0.69
SV (ml)	80.3 ± 2.6	84.1 ± 2.5	0.30	78.4 ± 3.5	82.9 ± 3.4	0.31
SV (ml/kgffm)	1.96 ± 0.1	1.99 ± 0.1	0.69	1.94 ± 0.1	1.96 ± 0.1	0.85
EDV (ml)	135.6 ± 4.1	136.2 ± 3.9	0.91	125.3 ± 5.2	128.1 ± 5.1	0.68
EDV (ml/kgffm)	3.2 ± 0.1	3.2 ± 0.1	0.67	3.03 ± 0.1	3.0 ± 0.1	0.85
ESV (ml)	54.9 ± 2.3	52.4 ± 2.2	0.37	46.7 ± 3.1	45.4 ± 3.0	0.72
ESV (ml/kgffm)	1.29 ± 0.04	1.21 ± 0.04	0.14	1.08 ± 0.06	1.05 ± 0.06	0.66
Workload (W)	—	—		43.6 ± 2.6	37.3 ± 2.5	0.08

Parameters were measured using magnetic resonance imaging (MRI) scans. Data are means ± SEM, adjusted for sex and age.

DBP: diastolic blood pressure; CO, cardiac output; EDV: end-diastolic volume; ESV: end-systolic volume; LVM: left ventricular mass; SBP: systolic blood pressure; SV: stroke volume.

**Table 4:** 24-hour ambulatory blood pressure (BP) monitoring among participants born post-term and at term.

		Term	Post-term	*P*-value
Mean heart rate (bpm)		72 ± 2	75 ± 2	0.29
Daytime	Diastolic BP (mmHg)	63.7 ± 1.3	63.9 ± 1.1	0.86
	Systolic BP (mmHg)	109.4 ± 1.6	108.7 ± 1.4	0.75
	Mean arterial pressure (mmHg)	80.2 ± 1.1	79.9 ± 1.0	0.89
Night time	Diastolic BP (mmHg)	53.8 ± 1.3	55.1 ± 1.2	0.44
	Systolic BP (mmHg)	100.9 ± 1.9	99.7 ± 1.6	0.63
	Mean arterial pressure (mmHg)	72.8 ± 1.3	72.4 ± 1.1	0.81
Night time dipping	Diastolic (%)	15.6 ± 1.9	13.7 ± 1.7	0.41
	Systolic (%)	7.8 ± 1.4	8.5 ± 1.2	0.69

Data are means ± SEM, adjusted for sex and age.

## Discussion

This is the first study to describe the cardiac function and exercise capacity of adolescents born post-term, who displayed a reduction in exercise capacity but no differences in cardiac function compared to term counterparts. This reduction in exercise capacity was not explained by changes in body composition, baseline physical activity, or left ventricular function.

As previously described, pre-pubertal children born post-term displayed reduced insulin sensitivity compared to those born at term^6^. Reduced insulin sensitivity has been linked to alterations in cardiac and peripheral cardiovascular function including altered endothelial function, hypertension and dyslipidemia, as well as an increased risk of cardiovascular pathology in later life^23,30^. Both SGA and preterm adolescents and young adults displayed reduced exercise capacity, and this has been correlated to changes in both cardiac and peripheral cardiovascular function^15–17^, in particular increased vascular thickness and left ventricular mass^14^. The reduction in exercise capacity noted in this study in subjects born post-term suggests there may be differences in cardiac function, peripheral vascular function, and/or altered muscle function. The lack of change in cardiac function indicates these changes are not central or cardiac, and we speculate that this may reflect an alteration in peripheral vascular or muscle function.

VO_2max_ is dependent not only on cardiac function but also on adequate oxygen diffusion and effective uptake of oxygen peripherally by the metabolically active tissues^31^. During exercise, there is increased systemic blood flow and hence capillary recruitment to improve oxygen extraction by skeletal muscle^32^. This is regulated by the endothelium and in particular nitric oxide^33^. Insulin is involved in activating the production of nitric oxide and reductions in insulin sensitivity reduce capillary vasodilation and recruitment, thereby reducing peripheral oxygen extraction^34^. As a reduction in insulin sensitivity in post-term pre-pubertal children has been documented^6^, this could be a factor reducing peripheral vascular perfusion in our older adolescent population and further investigations are required.

A study by Nadeau *et al*. has recently showed that insulin resistance may play an important role on the decreased exercise capacity in non-obese type 1 diabetic youth^20^. We did not directly measure insulin resistance in this cohort, but a previous study using intravenous glucose tolerant test and the minimal model methodology showed reduced insulin sensitivity in prepubertal children born post-term^6^. The lack of any observed difference in fasting insulin levels (which primarily measures hepatic insulin sensitivity) in this study is still consistent with a peripheral reduction in insulin sensitivity. Indeed, normal fasting insulin levels have been reported in preterm children who had evidence of a 40% reduction in insulin sensitivity using the minimal model approach^10^.

These finding potentially could be explained by a reduction in peripheral insulin sensitivity. As previously described, pre-pubertal children born post-term displayed at 34% reduction in peripheral insulin sensitivity compared to those born at term using intravenous glucose tolerance tests and the minimal model^6^. Importantly, fasting insulin levels (a measure primarily in hepatic insulin sensitivity) were the same in both post-term and term cohorts^6^. The similar fasting insulin levels are consistent with the current study. We understand this was a limitation of our study and future research looking into peripheral insulin sensitivity and cardiac function in this population should be explored.

Our relatively small sample size is a limitation of our study as small differences in outcomes might have been missed. However, we contend that cardiac MRI with submaximal exercise provides extremely precise and reproducible data, and it is unlikely any clinically-relevant differences were missed. The control group was also heterogeneous with approximately 30% being siblings of post-term participants. Self-reported activity is less reliable than quantified measures of physical activity (e.g. accelerometers), but this was the only approach available for this study. While this is a limitation, the adopted questionnaire does provide an estimate of physical activity levels, and this method has been used previously in published clinical studies^6,25,26^.

While the current clinical relevance of the lower exercise capacity observed in adolescents born post-term is unclear, it is well established that lifestyle and aging can lead to a more marked reduction in VO_2max_^35^. Therefore, over time this difference in VO_2max_ may become greater (as lifestyle tend to change into adulthood), being potentially associated with an increased risk of diseases linked to a sedentary lifestyle. Thus, we suggest that the maintenance of an active lifestyle may be particularly important for those born post-term.

In summary, our study has provided some evidence of a reduction in exercise capacity in adolescents born post-term despite similar central cardiac function as those born at term. This difference in exercise capacity could be attributed to differences in peripheral vascular function and suggest further studies testing peripheral blood flow and compliance need to be conducted.

## Data Availability

The clinical data cannot be made available in a public repository due to strict conditions of the ethics approval of our study. Nonetheless, anonymized and unidentifiable data would be made available to other investigators upon request. For this purpose, anyone interested should contact the senior author (P.L.H.).
